# Breast phantom with silicone implant for evaluation in conventional mammography

**DOI:** 10.1120/jacmp.v12i1.3340

**Published:** 2010-09-20

**Authors:** Fábio A. R. Silva, Luíza F Souza, Carlos E. G. Salmon, Divanizia N Souza

**Affiliations:** ^1^ Departamento de Física Universidade Federal de Sergipe São Cristovão Sergipe Brazil; ^2^ Departamento de Física Faculdade de Filosofia, Ciěncias e Letras de Ribeirão Preto, Universidade de São Paulo São Paulo Brazil

**Keywords:** breast phantom, silicone prostheses, mammography, quality assurance

## Abstract

With the increased incidence of cancer and a similarly increased number of surgeries for insertion of silicone breast implants, it is necessary to assess the effect of such material within the breast tissue, particularly in mammography, because of the reduction in the power of breast cancer diagnosis. In this work, we introduce a breast phantom with silicone implants in order to evaluate the influence of the implant on the visibility of the main mammographic findings: fibers, microcalcifications and tumor masses. In this proposed phantom, the breast tissue was simulated using gel paraffin. In the optical density of phantom mammograms with implants, a reduction in breast tissue visibility was seen corresponding to 23% when compared to a phantom without silicone implants. This poor visibility was due to the X‐ray beam scattering on silicone material; this effect produced a loss of visibility in the areas adjacent to the implant. It is expected that the proposed phantom model may be used as a device for the establishment of a technical standard for these types of procedures.

PACS number: 87.59 E

## I. INTRODUCTION

Surgeries for breast reconstruction after mastectomy and for breast augmentation have become two of the most common plastic surgeries performed in the world. In these procedures, a variety of implants are used to simulate the natural breast both in appearance and texture.^(^ ^1^ ^)^ In addition, the higher incidence of breast cancer in the world has increased interest in knowledge on this subject by society in general.

The National Academy of Science published a report showing that, according to epidemiological evidence, in 1999 the United States had more than 1.5 million adult women of all ages with silicone breast prostheses. This report also predicted that some of these women would develop connective tissue diseases, as well as other diseases. However, evidence suggests that the diseases or conditions in women with implants are as common as in women without implants.^(^ ^2^ ^)^

According to Silverstein et al.,^(^ ^3^ ^)^ anterior breast tissue is generally seen better with displacement mammography, whereas posterior breast tissue is seen better with compression mammography. These authors also suggested that better films are generally obtained when the implant is in the subpectoral position rather than subglandular.

In mammography, an accurate diagnosis often depends on the visibility of small low‐contrast objects within the breast image. Extreme density differences produced by silicone prostheses result in a low radiographic density due to the radiopaque characteristics of these implants.^(^ ^4^ ^)^ In addition, the implants promote inadequate breast compression, which negatively influences the image quality.

Currently, mammography remains the most important monitoring method for breast cancer diagnosis, and numerous studies have been published assessing or improving quality control in this type of examination. However, there is no data in the literature related to quality control of the image used in mammograms of women with silicone implants. For such patients, the technique applied in mammography is different from the one used on breasts without prostheses. In the later case, examinations are performed with mammographic equipment operating in the manual mode without automatic exposure control due to the presence of nonequivalent breast tissue and the difficulty of breast compression.^(^ ^5^ ^)^

More accurately, the technical aspects which describe a good image are basically determined by five parameters: high spatial resolution, high image contrast, low image noise, ideal optical density within the range of 1.0 to 2.5, and the nonexistence of artifacts.^(^ ^6^ ^)^ It is essential to remember also that these aspects should never be separated from the amount of the absorbed radiation dose received by the patient.

In the United States, mammography is regulated by the federal government through the mechanism of mandatory certification issued by the Food and Drug Administration (FDA) and other accreditation institutions. In the American Mammography Certification Program, one of the requirements to demonstrate image quality is the viewing of four fibers, three groups of calcifications and three simulations of tumor masses in an approved phantom. In Brazil, according to Resolution No 1016, the minimum requirements to be viewed in a breast phantom image, as adopted by the ACR, are the presence of test structures specified by the American program and a background optical density of 1.40 OD.^(^ ^7^ ^)^

Given the importance of careful evaluation of the parameters used for breast imaging, we produced a breast phantom with a silicone implant for the evaluation of physical and clinical parameters in screen‐film mammography. The new phantom allowed the assessment of image quality in terms of test structures and the optical density, verified the influence on the image of silicone prostheses in relation to breast tissue, and served as a guidance device for finding the best parameters of kilovoltage and mAs when performing mammography. The proposed phantom should assist in the development of methods for procedures for improved quality and safety in mammographic examinations.

## II. MATERIALS AND METHODS

The base material used in making the simulator was obtained from a mixture of gel paraffin added to self‐polymerizing acrylic. The mix of these two compounds resulted in a substance that provided similar characteristics, in terms of optical background density, to those measured in standard mammograms.

The female breast phantoms had a semi‐spherical appearance with a diameter of 12 cm for the base, a height of 5.5 cm and a volume of approximately 600 mL. The soft breast compression during the mammography reduces the phantom thickness by up to 10%.

Artifacts which simulate the main findings in mammography and a 105 mL silicone implant were inserted into the base material. Nylon yarn having sizes from 0.40 to 1.55 mm were used to simulate fibers; ground porcelain with particle diameters between 0.20 mm and 0.90 mm was used to simulate microcalcifications, and nylon masses with spherical diameters in the range of 1.0 mm to 5.0 mm and thicknesses between 0.27 mm and 2.5 mm were used to simulate tumor‐like masses, in agreement with the standards specified by the Brazilian legislation.

Four samples of phantom were made: the first sample only contained the material base; the second sample contained the material base and the silicone implant (105 mL) in order to verify the relationship between optical density of the image in the area of the implant and in the region that simulates breast tissue; the third sample contained the base material with fiber artifacts, microcalcification artifacts and tumor‐like artifacts inserted; and, in the fourth sample, a silicone breast implant was inserted into the base material containing the inclusions.

As the proposed phantom has both a texture and mobility similar to the ones used in breast implants, the use of mammography in the automatic mode was not possible due to the risk of implant rupture during machine compression and overexposure of the film due to high‐intensity beam selected by the automatic exposure control.

The phantom images were obtained using model Graph‐Mammo AF mammographic X‐ray equipment (VMI Industria e Comercio Ltda, Lagoa Santa, Brazil) with the following conditions: manual mode with 25 kVp and mAs varying in the range of 40–200 mAs. The kVp value chosen is the standard used for mammographies with similar configurations to the phantom constructed. Screen‐film mammography films (Konex Industria e Comercio Ltda, Sao Paulo, Brazil) of 18 by 24 cm were used. Compensation or variation in density control or magnification was not used in the images. The images were processed by an automatic processor Kodak X‐OMAT 3000RA (Eastman Kodak Company, Rochester, NY). A breast compression device was used in contact with the simulator and an antigrid diffuser. The films were properly masked and read by medical physicists on a dedicated mammography view box (model KO‐NM4, Konex) with luminance levels of at least 3000 units. The optical densities were analyzed at each point of interest in the image using a model 34 densitometer (X‐Rite, Grand Rapids, MI). The images were then qualitatively analyzed by a radiologist. All equipment and accessories were dedicated exclusively to mammography and manufactured in Brazil.

## III. RESULTS & DISCUSSION

The final results of quality and functionality of the proposed simulator were obtained from the values of OD read at each point in the images and by means of analyses of the spatial resolution shown by the artifacts displayed in the films. The optical density values and the details in the image were found by reading 10 images from the same reference parameters.

**Figure 1:**
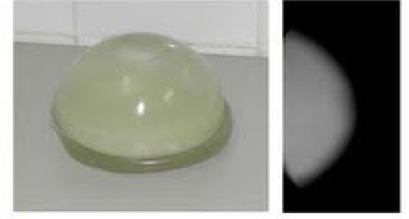
The sample with base material and its mammographic image.

Figure 1, on the left, shows an image of the phantom which only contained the base material, and on the right, a mammogram obtained from this phantom. The mammographic image presents an optical background density of 1.46 OD.

For determination of the appropriate optical density, the sample which only contained the base material was exposed to a fixed value of 25 kVp and a range of 40 mAs to 200 mAs, which was the range determined by the thickness and density of the sample and clinically feasibility. Table 1 shows the optical density values obtained from the images of the phantoms with and without silicone implants by each technique selected.

**Table 1:** Relationships between the selected technique and the optical density value for the determination of the appropriate optical background density.

*mAs*	*No Implant Optical Density*	*With Implant Optical Density*
40	0.47	0.34
60	0.66	0.48
80	0.91	0.75
100	1.33	1.04
120	1.46	1.12
140	1.47	1.14
160	1.51	1.39
180	1.94	1.61
200	2.27	2.19

According to the Table, the technique using 25 kVp and 120 mAs showed the most appropriate optical density and served as the exposure parameter of this sample in later stages of the study.

**Figure 2:**
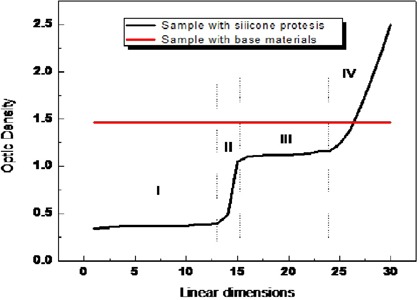
Behavior in terms of optical density measured in sample with silicone prosthesis and sample with base material alone.

**Figure 3:**
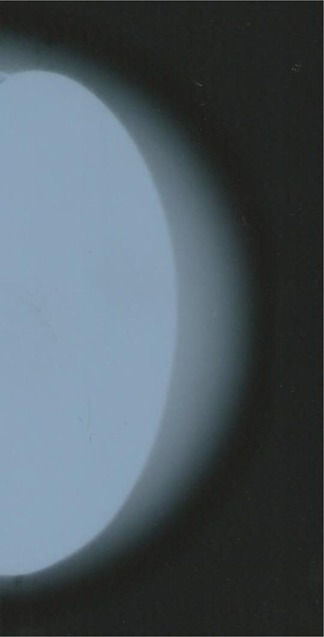
Image of the phantom containing only the base material and the silicone breast implant.

Figure 2 shows the behavior of optical density as a linear fit from the image corresponding to the sample that only contained the base material and which simulated breast tissue, and from the image corresponding to the sample in which the prosthesis was inserted into the base material. Differences in optical densities caused by X‐ray beam interactions in the sample containing the prosthesis can be explained by describing the four regions observed in this mammogram. The first region (region I) is the region which corresponds to a sharp reduction in optical density due to the material of the implant. A transition region can be observed between the prosthesis and the material that simulated the breast tissue (region II). In the third region (region III), a gradient of optical density was found between the prosthesis and the outer limits of the base material. In this band the optical density was reduced from 1.45 to 1.12 on average, a reduction of 23% in the power of breast tissue visualization. In the last region (region IV), the darkest part of the film is observed on the direct exposition. In Fig. 3 we can see the intense brightness caused by the interaction of radiation on the silicone breast implant.

**Figure 4:**
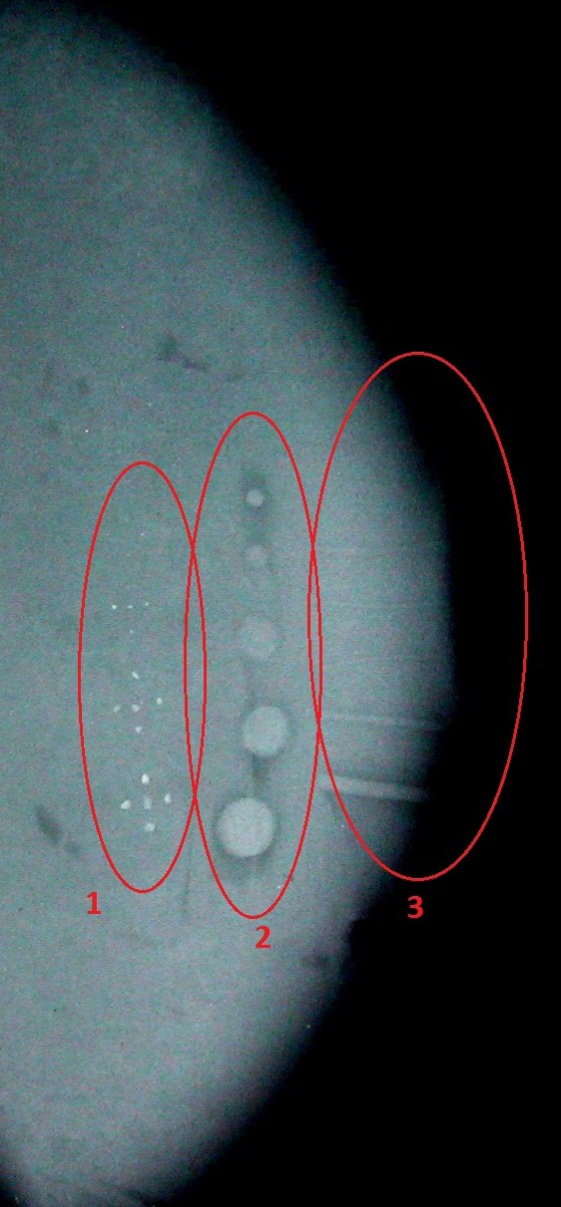
Image of the base material containing inserted artifacts: microcalcifications (detail 1), tumor masses (detail 2) and fibers (detail 3).

An image of the phantom sample containing the artifacts can be seen in Fig. 4. These artifacts were used to simulate the main mammographic findings and include: four sets of microcalcifications, five tumor‐like masses and five fibers. However, it was only possible to visualize three sets of microcalcifications (detail 1), five tumor masses (detail 2), and four fibers (detail 3) in the image of this phantom sample.

**Figure 5:**
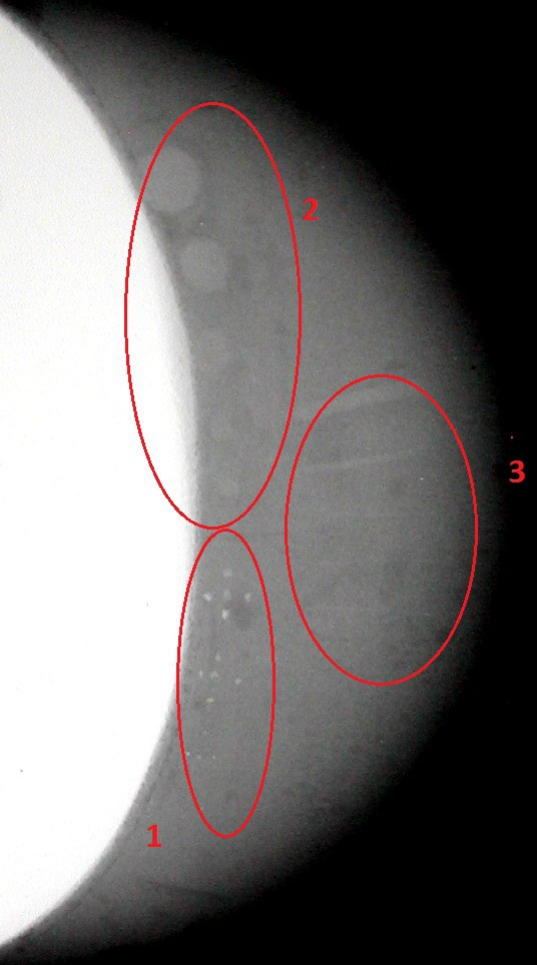
Image of the proposed phantom containing the silicone implant and the inserted artifacts: microcalcifications (detail 1), tumor masses (detail 2) and fibers (detail 3).

Figure 5 shows the fourth phantom image containing both the prosthesis and the artifacts which simulated the main mammographic findings. The display of the test structures suffered a loss in sharpness because of the scattering of radiation within the prosthesis. Compared to Fig. 3, the number of artifacts that could be seen were reduced; only two sets of microcalcifications (detail 1), four tumor‐like masses (detail 2), and two fibers (detail 3).

Table 2 shows the visualization of the structures for each value of mAs selected. The data show that most objects were visualized in the images exposed using 120 mAs and 130 mAs. The number of structures visualized at 140 mAs decreased by one in each group.

**Table 2:** Relationships between the value of mAs and the number of structures visualized within the proposed phantom.

*Structures*	*80 mAs*	*100 mAs*	*120mAs*	*130 mAs*	*140 mAs*
Fibers	2	2	5	5	4
Microcalcifications	2	3	4	4	3
Tumoral Mass	5	5	5	5	4

## IV. CONCLUSIONS

We studied the effect of silicone prostheses in mammography images and proved that the insertion of a silicone prosthesis into the breast caused a diminished view of breast tissue areas. This could lead to a reduction in the accuracy of breast cancer diagnoses in breasts containing silicone implants and could, therefore, be a risk to health. A reduction in the spatial resolution and image contrast was highlighted, which was due to the different characteristics of the silicone implant and breast tissue materials, beam scattering in the areas adjacent to the implant, and inadequate breast compression which allowed the overlapping of structures.

The routine for performing mammography in breasts with silicone implants should not be the same as for that in breasts without implants. In this study, it was noticed that breasts with implants should not be compressed as much as in the mammography of breasts without implants, and the radiology procedure should be performed with the device operating in manual mode. Changing other mammography screening parameters can also cause differences in mammography procedures. For example, time exposition kilovoltage modification can cause changes in the dose of radiation prescribed for the procedure. Thus, besides showing the influence of prosthetic silicone breasts on mammography, it is expected that the proposed phantom model may be used as a parameter for the establishment of a technical standard for these types of procedures.

It can be seen by analyzing the images just how much the prosthetic material reduced the view of the base material which simulated the breast tissue. A reduction of almost 30% was observed due to scatter, which created a gradient of brightness from the area adjacent to the prosthesis to the outer limits of the base material. However, even with the loss of sharpness due to the prosthesis, it was possible to observe most of the artifacts, thus illustrating how effective this simulator was for this particular application.

Even with the possibility of improving image quality through quality control aided by the use of the phantom, some aspects of the breast, especially those related to the posterior parts of the breast, are poorly seen because the implant limits the view of this region.

## ACKNOWLEDGEMENTS

This work was partially supported by Conselho Nacional de Desenvolvimento Científico e Tecnológico (CNPq), Coordenação de Aperfeiçoamento de Pessoal e Nível Superior (CAPES), Brazil.
